# Past, present, and future of Phase 3 vaccine trial design: rethinking statistics for the 21st century

**DOI:** 10.1093/cei/uxae104

**Published:** 2024-11-21

**Authors:** Leila Janani, Rachel Phillips, Ellie Van Vogt, Xinxue Liu, Claire Waddington, Suzie Cro

**Affiliations:** Imperial Clinical Trials Unit, School of Public Health, Imperial College London, London, UK; Imperial Clinical Trials Unit, School of Public Health, Imperial College London, London, UK; Imperial Clinical Trials Unit, School of Public Health, Imperial College London, London, UK; Oxford Vaccine Group, Department of Paediatrics, University of Oxford, Oxford, UK; NIHR Oxford Biomedical Research Centre and Oxford University Hospitals NHS Foundation Trust, Oxford, UK; Department of Infectious Diseases, Imperial College NHS Healthcare Trust, St Mary’s Hospital, London, UK; Imperial Clinical Trials Unit, School of Public Health, Imperial College London, London, UK

**Keywords:** vaccine, randomized clinical trials, adaptive designs, Phase 3, estimand

## Abstract

Vaccines are crucial for protecting health globally; however, their widespread use relies on rigorous clinical development programmes. This includes Phase 3 randomized controlled trials (RCTs) to confirm their safety, immunogenicity, and efficacy. Traditionally, such trials used fixed designs with predetermined assumptions, lacking the flexibility to change during the trial or stop early due to overwhelming evidence of either efficacy or futility. Modern vaccine trials benefit from innovative approaches like adaptive designs, allowing for planned trial adaptations based on accumulating data. Here, we provide an overview of the evolution of Phase 3 vaccine trial design and statistical analysis methods from traditional to more innovative contemporary methods. This includes adaptive trial designs, which offer ethical advantages and enable early termination if indicated; Bayesian methods, which combine prior knowledge and observed trial data to increase efficiency and enhance result interpretation; modern statistical analysis methods, which enable more accurate and precise inferences; the estimand framework, which ensures the primary question of interest is addressed in a trial; novel approaches using machine learning methods to assess heterogeneity of treatment effects; and statistical advances in safety analysis to evaluate reactogenicity and clinical adverse events. We conclude with insights into the future direction of vaccine trials, aiming to inform clinicians and researchers about conventional and novel RCT design and analysis approaches to facilitate the conduct of efficient, timely trials.

## Teaser

This paper provides an overview of the evolution of Phase 3 vaccine trial design and statistical analysis methods from traditional to more innovative contemporary methods.

## Introduction

Vaccines have a long history of protecting human health [1, 2] and continue to be indispensable for effectively controlling and containing infectious diseases. Structured vaccine programmes and systematic vaccination campaigns are now crucial to global health efforts [3]. However, these are only possible following well-conducted development programmes, including clinical trials to support the approval of new or modified vaccines.

The safety, immunogenicity and efficacy of vaccines are usually determined in prospective, double-blind, randomized controlled trials (RCTs) [4]. Confirmatory Phase 3 RCTs, which focus on these aspects in large populations, are the final stage before regulatory approval for most vaccines.

Vaccine trials involve unique design challenges compared to other therapies. Vaccines are typically administered in 1–3 doses over a short period of time to healthy individuals who may never contract the disease they are designed to prevent. This necessitates a high standard for safety and the benefit-risk assessment [5, 6]. Moreover, vaccine trials typically require large sample sizes to ensure a sufficient number of individuals contract the disease, enabling the detection of the true effect of the vaccine. Recruiting participants over a short period, especially for emerging infectious diseases, poses challenges in identifying high-risk populations to achieve the necessary number of cases within a specific timeframe. Additionally, heterogeneity in the study population can impact vaccine efficacy and is crucial to understand [7].

Conventionally, Phase 3 RCTs adopt a parallel, two-group, fixed-allocation-ratio design, analysed with frequentist statistical methods at a single prespecified endpoint. However, clinical trials require a multidisciplinary team effort, with statisticians and trial methodologists playing increasingly crucial roles in designing more efficient Phase 3 trials to answer questions faster. Global pandemics like COVID-19 have highlighted the need for use of more efficient trial designs and analyses. For example, five large Phase 3 RCTs of COVID-19 vaccines incorporated adaptive trial methods, which enabled the trials to stop early when vaccine efficacy was declared [8].

This article provides an overview of innovative clinical trial designs and analysis methods that are applicable to vaccine trials. It begins with an exploration of the history of vaccine trials, reviews both traditional and contemporary trial designs, discusses the use of estimands and new analytical approaches, and concludes with insights on the future direction of vaccine trials.

In the following sections, we will use common statistical terms in the design and analysis of clinical trials. Table 1 provides a glossary of these terms [9, 10].

**Table 1:** glossary of common statistical terms

Hypothesis	In statistics, a hypothesis is a statement about the study population, typically proposing that a population parameter (e.g. treatment effect) takes a specific numerical value or falls within a certain range.
Null hypothesis	The null hypothesis is a statement that the parameter of interest (e.g. treatment effect) takes a particular value. In clinical trials, this usually means that the treatment effect on the outcome compared to the control is zero. It is the hypothesis we aim to test and potentially reject.
Alternative hypothesis	The alternative hypothesis states that the parameter of interest falls in some alternative range of values. In clinical trials, this usually represents the possibility that the treatment has an impact on the outcome. It is what the trial aims to demonstrate, i.e. that the new intervention is superior (or different) compared to the control.
Types I II errors rates	Two potential errors are commonly recognized when testing a hypothesis, called Types I and II errors. Type I occurs when we wrongly reject the null hypothesis when it is actually true, concluding that the treatment has an effect when it does not (i.e. a false-positive result). The probability of making a Type I error is called alpha (α). Type II error happens when we fail to reject the null hypothesis when the alternative hypothesis is true, meaning we miss detecting a true treatment effect (i.e. a false-negative result). The probability of making this error is called beta (β).
Power	The power is the probability of correctly rejecting the null hypothesis when it is false, i.e. detecting a true treatment effect. Power is calculated as (1 − β) and is typically set at 80% or 90%, indicating the trial has a high 80% or 90% chance of detecting a real effect if it exists.
*P*-value	*P*-value is the probability that the test statistic equals the observed value or a value even more extreme assuming that the null hypothesis is true. A smaller *P*-value suggests stronger evidence against the null hypothesis.
Significance level	The significance level is the threshold for rejecting the null hypothesis. If *P*-value is less than or equal to this significance level, we reject the null hypothesis, indicating evidence of a treatment effect. In practice, the most common significance level used is 0.05.
Confidence intervals	A confidence interval describes the uncertainty around the estimated treatment effect based on the trial’s sample. A 95% confidence interval means that if we were to repeat the trial 100 times, about 95 of those intervals would contain the true population treatment effect. Confidence intervals offer insight into the precision of the effect estimate and are typically reported alongside *P*-values in trial results.
Nuisance parameters	Nuisance parameters in statistics refer to parameters that are not of direct interest but must be accounted for in hypothesis testing and models. A classic example of nuisance parameters is when estimating the mean of a normal distribution, where the variance is known as a nuisance parameter if only the mean is of interest.
Interim analysis	Refers to any examination of data obtained from subjects while a trial is ongoing. This analysis is not limited to formal between-group comparisons.

## History of vaccine trials

Although the practice of vaccination began with Edward Jenner’s 1796 experiment involving exposure to milkmaids’ cowpox lesions to confer immunity against smallpox, it was not until the 20th century that randomized controlled trials (RCTs) became the gold standard in clinical research [11].

The first controlled clinical trial of the modern era is often attributed to James Lind’s scurvy experiments in 1747, which involved dividing 12 sailors into six pairs and giving each pair a different dietary supplement [3]. The UK Medical Research Council (MRC) conducted the first double-blind controlled trial, though non-randomized, for patulin in treating the common cold in 1943–44. The first RCT was conducted by the MRC in 1946, testing streptomycin for pulmonary tuberculosis [12].

A significant advancement in vaccine trial design came in the 1950s with the polio vaccine trials. These landmark trials involved over a million children and utilized both randomized placebo-controlled trials and ‘observed control’ designs, setting a precedent for large-scale Phase 3 trials [13].

**Figure 1:**
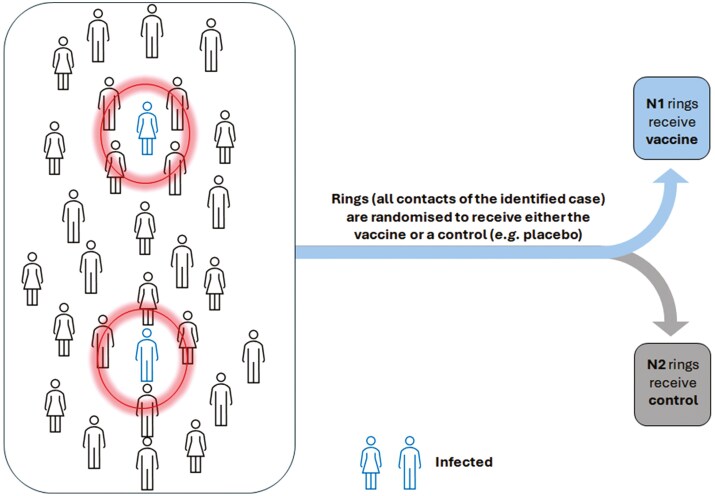
ring vaccination cluster randomized trial. Each ring, shown by circles, is formed around an index case (the infected individual) and is treated as a cluster in the trial, with each cluster randomly assigned to either the vaccine or control group

More recent public health emergencies, including the Ebola outbreak, provided a challenging environment requiring the incorporation of more flexible designs in the evaluation of vaccines. A notable example is the use of a ring vaccination design [14] to assess the rVSV-ZEBOV vaccine [15]. A ring vaccination design involves identifying contacts and contacts of contacts of a newly identified case to form a ring, and these rings are randomized as part of a cluster-randomized trial or with individual randomization within rings. Key advantages of the design include its flexibility in taking vaccinations to where transmission is occurring and following the epidemic as it progresses, as well as its ability to target high-risk participants to increase power [16]. Figure 1 illustrates how ring vaccination trial work in practice.

The stepped wedge cluster trial is another design used to test the recombinant vesicular stomatitis virus Ebola vaccine (rVSV ∆ G-ZEBOV-GP) in Sierra Leone [17]. In the STRIVE trial, the population consisted of geographically distinct clusters, such as clinics or hospitals, that were randomly and sequentially assigned to vaccination over several time periods. By the end of the study, all participants had received the intervention. This design is particularly desirable when vaccination cannot be introduced to all clusters simultaneously due to logistical or financial constraints. It also offers the ethical advantage of not withholding vaccines from unvaccinated clusters while they serve as control groups [18, 19].

The 2019–2021 coronavirus disease (COVID-19) pandemic posed a tremendous challenge to global health systems and triggered the unprecedented rapid development of several vaccines. In response to the urgent need for population immunization, it became crucial to conduct efficient, swift, and well-designed trials to test these vaccines, some of which we explore further below [3].

## Phases of clinical trials and traditional trial designs

Vaccine development begins with discovery and laboratory research. Researchers typically conduct pre-clinical assessments in animals to evaluate immune response potential. Candidate vaccines then progress through multiple clinical trial phases involving humans [20]. Typically, human assessments begin with Phase 1 trials, which evaluate various doses, focussing on safety and reactogenicity and providing preliminary immunogenicity assessments. These trials are usually open-label and non-randomized, with descriptive analysis involving a small number of participants (e.g. < 20).

Phase 2 trials are then conducted with a larger and more specific population to further assess safety and immunogenicity and to identify the optimal dose and schedule. Phase 2 trials typically involve parallel group comparisons with placebo or active control groups and use inferential statistics to evaluate outcomes [21, 22].

Phase 3 trials are larger randomized controlled trials designed to measure the preventive efficacy of the vaccine against the disease of interest and provide the pivotal data needed for marketing approval alongside more extensive safety and immunogenicity data. Vaccine efficacy (VE) is defined as “the percentage reduction in the incidence of disease or infection among those vaccinated compared to unvaccinated individuals” [23]. Demonstrating VE through a Phase 3 trial is crucial for obtaining licensure and informing policy-makers about potential vaccine uses [22, 24].

After successful completion of Phase 3 trials and following licensure of the product, Phase 4 studies, also referred to as postmarketing surveillance studies (PMS) are used to continue to monitor the vaccine for safety and effectiveness in the population. The main reason for undertaking Phase 4 studies is to monitor vaccine effectiveness and document the less frequent adverse reactions [22, 25].

Traditionally, Phase 3 vaccine trials have employed a fixed design with predetermined assumptions established during the planning phase, allowing no flexibility for modifications during the trial. However, these design assumptions can often be uncertain or incorrect, potentially leading to a trial design that fails to adequately address the research questions [26]. Additionally, a fixed design can impede rapid clinical decision-making, which is crucial during emergencies such as the Ebola or COVID-19 outbreaks [27, 28].

## Innovative and adaptive designs

In recent years, researchers have developed more efficient adaptive vaccine trials to reduce development timelines, use fewer resources, and provide more accurate estimates of endpoints. These RCTs aim to deliver effective vaccines to the public faster and at a lower cost [29, 30].

An adaptive design is defined as “a clinical trial design that allows for prospectively planned modification to one or more aspects of the design based on accumulating data from subjects in the trial” [31]. Modifications can include dropping ineffective treatment arms, modifying the population, or reducing sample size. Adaptive designs offer ethical advantages over fixed designs, such as terminating trials early if there is overwhelming evidence of efficacy or if showing efficacy is unlikely, minimizing risk to participants [32, 33].

Adaptive designs can be applied across all phases of clinical research, from early-phase dose escalation to late-phase confirmatory trials. Common early-phase adaptive approaches include the Continual Reassessment Method (CRM) [34] and Seamless Phase 1/2 designs [35]. This article focuses on late-phase adaptive designs, emphasizing that their application to individual vaccine trials necessitates context-specific considerations and may require additional methodological development.

Using the FDA’s categorization [31], we now introduce different types of adaptive designs.

## Types of adaptive designs

1. Group sequential designs

Group sequential designs are two-arm RCTs which incorporate prospectively planned interim analyses using statistical hypothesis tests at predefined intervals with predetermined criteria for stopping the trial early. Such designs may include rules for stopping a trial when there is sufficient evidence of efficacy to support regulatory decisions or when evidence suggests the trial is unlikely to demonstrate efficacy, a scenario referred to as stopping for futility. Group sequential designs can reduce the expected sample size and have the potential to shorten the overall trial duration and accelerate the approval of new therapies [31, 32].

**Figure 2:**
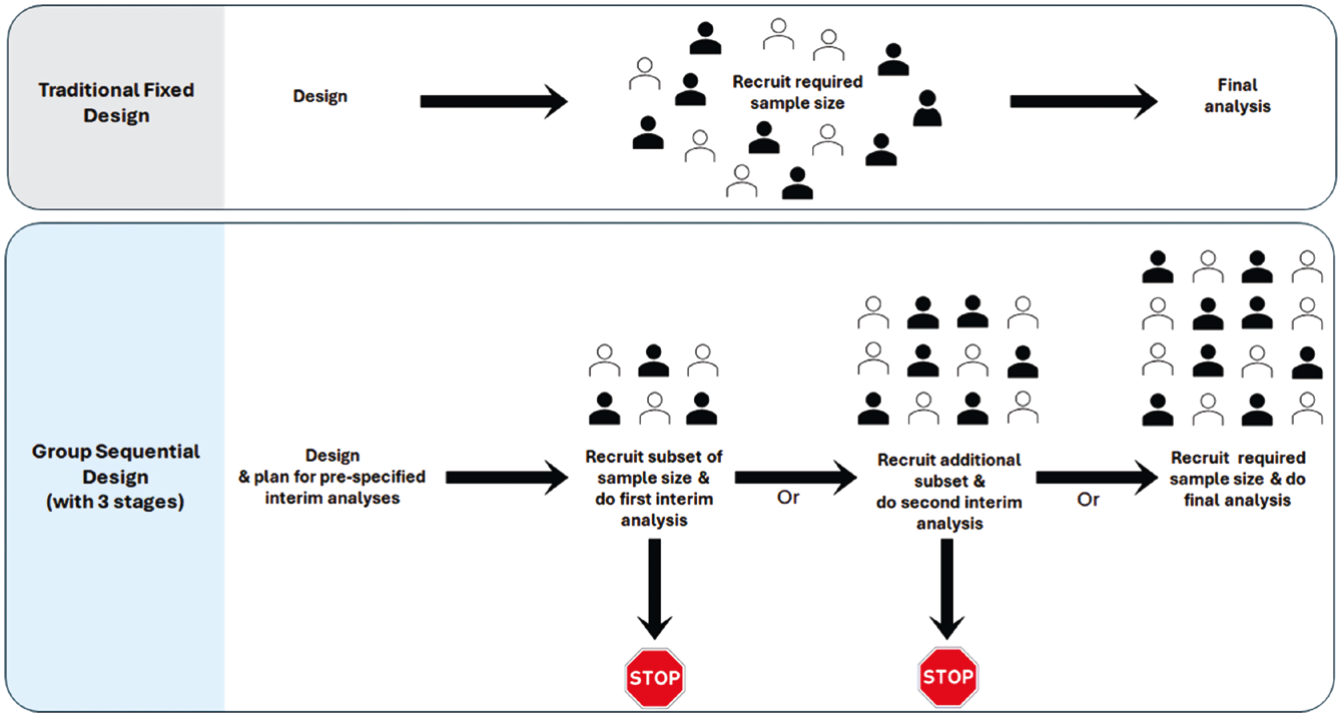
comparison of traditional fixed design and group sequential design with three stages. Group sequential design can include various numbers of stages; the figure illustrates an example with three stages. The filled and outlined represent participants assigned to the two arms of the study

Figure 2 illustrates the traditional fixed design compared to the group sequential design, which includes two prespecified interim analyses and one final analysis, comprising a total of three stages.

Under the frequentist framework, repeatedly statistically testing the null hypothesis (H0, of no treatment effect) at a traditional significance level α throughout the trial (typically 0.05) can inflate the overall chance of declaring a treatment as effective when it is not (Type I error rate inflation) beyond α. To address this, in group sequential designs, the statistical significance threshold (α) is adjusted at each interim test, which in practise means the statistical significance threshold (α) is adjusted downward at each interim analysis compared to what would be used in a trial without repeated testing. There are various methods for doing so; for instance, the O’Brien-Fleming approach [36] requires very compelling early results to justify stopping the trial for efficacy (i.e. a very small significance threshold α used in early interim analysis and as the trial progresses, the significance threshold α becomes less conservative, so it’s easier to reach significance later). Alternatively, methods like the Pocock approach [37] use the same adjusted significance level at each interim analysis, and require less persuasive early results, thus have a higher chance of early stopping. For example, with two interim analyses and a final analysis (three stages), if we obtain a *P*-value of 0.018 at the second interim analysis, under the O’Brien-Fleming approach we would continue the trial because 0.018 > 0.0151 (the O’Brien-Fleming significance level at that point). However, using the Pocock approach, we would stop the trial for efficacy since 0.018 < 0.021 (the Pocock significance level), indicating statistical significance.

The Phase 3 trial of a recombinant glycoprotein 120 vaccine to prevent HIV-1 infection conducted by the rgp120 HIV Vaccine Study Group [38] was designed using a group sequential approach with one prespecified interim efficacy analysis scheduled 40 months after the initiation of the trial. Five large Phase 3 RCTs of COVID-19 vaccines also used sequential trial methods which enabled the trials to stop early when vaccine efficacy was declared, speeding up the delivery of efficacious COVID-19 vaccines globally [8].

In general, for vaccine studies with rapid recruitment periods, group sequential designs may not significantly impact the sample size. However, as demonstrated by the examples above, there are circumstances where a group sequential design is appropriate and can save time and costs in identifying efficacious vaccines.

2. Multi-arm multi-stage design

**Figure 3:**
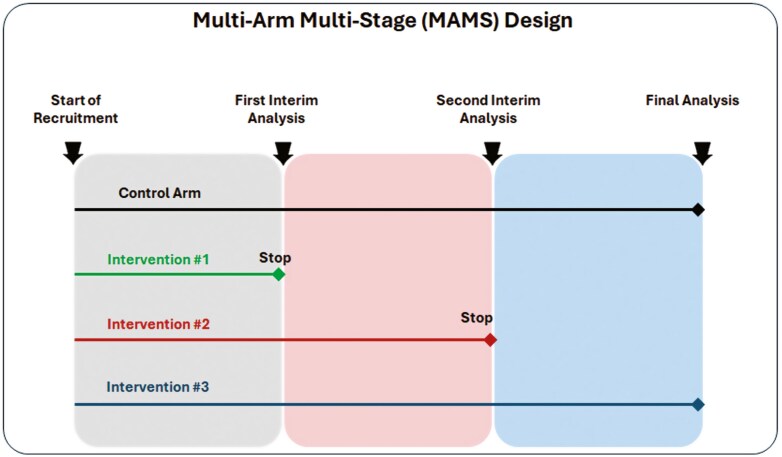
multi-arm multi-stage (MAMS) design with 4 arms and 3 stages (two interim analyses and one final analysis)

A multi-arm, multi-stage (MAMS) design extends the group sequential design to allow multiple treatment arms to be compared to a shared comparator, such as a control group (or placebo) [39]. Treatment arms that show adequate performance at planned interim analyses based on accumulated data are retained for further testing, whilst those that do not can be dropped early. The criteria for continuing or dropping treatments at interim analyses is prespecified and requires careful statistical consideration [40–42]. Typically, a relaxed α is used at the interim stage with high power to protect against incorrectly discarding an effective treatment arm early. MAMS trials have been used in the context of early phase HIV vaccine trials [43] but they also offer substantial efficiency gains over conducting a series of single-stage, two-arm Phase 3 trials. Such gains have been realized in multiple non-vaccine Phase 3 settings [44]. When there are multiple potential interventions available for testing, they can be advantageous to address multiple research questions simultaneously. Figure 3 illustrates MAMS design with 4 arms and 3 stages (two interim analyses and one final analysis).

3. Sample size re-estimation

Calculating sample size is a crucial step in trial design to ensure a trial includes an adequate number of participants to detect effect sizes of interest. But this relies on assumptions about the anticipated treatment effect(s) and variability of the outcome measurement. These assumptions are typically based on data from previous studies, but data from the current trial may also be used to revisit initial assumptions during the trial to provide the trial with a better chance of demonstrating the effects of interest [45].

Sample size re-estimation (SSR) is an adaptive approach allowing for changes in the trial’s required sample size or required number of events (for time-to-event outcomes) after an interim analysis. Pre-specification of the sample size design parameters that will be re-estimated during the trial, for example the anticipated treatment effect(s) and/or nuisance parameters such as the outcome variability (i.e. standard deviation) is required.

Blinded SSR refers to estimating nuisance parameters through non-comparative analysis, whether with pooled outcome data without knowledge of treatment assignment (like the variance of continuous outcomes in pooled data) or with knowledge of treatment assignment (like the probability of a binary outcome on the control arm) [31]. This approach eliminates the need to adjust the sample size based on the estimated treatment effect.

Blinded SSR has minimal to no impact on the Type I error probability. However, unlike adaptations that rely on pooled data (without knowledge of treatment assignment), if SSR uses treatment assignment information, additional steps are needed to maintain trial integrity.

For instance, a two-arm, participant-blinded RCT on rabies vaccine effects used blinded SSR, with sample size re-estimation planned based on three nuisance parameters [46].

In cases of considerable uncertainty about treatment effect size, unblinded SSR can be planned based on comparative interim analysis results and estimated treatment effect using various methods [47–50].

In vaccine trials, where assumptions about efficacy and incidence rates are often uncertain, adapting the sample size based on accumulating data may enhance the likelihood of demonstrating the effects of interest.

4. Adaptive population enrichment

With the rapid advancement of genomic technology and precision medicine, interest has grown in enhancing treatment effectiveness for specific trial subpopulations. Early-phase trials may indicate heterogeneity in treatment response. In such cases, adaptive population enrichment (APE) designs, which facilitate the selection or enrichment of populations, may be advantageous. Subpopulations can be defined by demographic characteristics, or genetic or pathophysiological markers related to the drug’s mechanism of action [31].

A trial with an APE design, tests the treatment effect in the broad target population and specific subpopulations with adequate statistical power. Initially, participants from a broad population are enrolled and randomized until an interim analysis is conducted. Based on prespecified criteria, the interim analysis determines whether to continue enrolling the general population or restrict enrolment to a subpopulation showing treatment benefits. Targeted subpopulations must be defined in advance. Statistical hypothesis testing must account for the multiplicity of testing hypotheses across multiple populations and time points.

**Figure 4:**
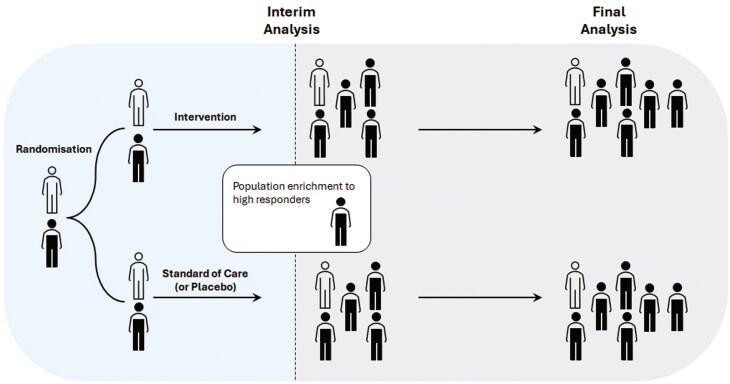
adaptive population enrichment design with 2 arms and 2 potential subpopulations. Filled indicates one subpopulation (e.g. the biomarker-positive subgroup), while outlined represents the second subpopulation (biomarker-negative subgroup)

Figure 4 illustrates APE design with two potential subpopulations.

Su et al. [51] illustrate an adaptive population-enrichment design strategy for an event-driven vaccine efficacy trial. This design provided adequate power to test a vaccine across two subpopulations, where concerns of heterogeneity in response had been identified in initial Phase 1/2 trial results.

The TAPPAS trial, a randomized, multinational, open-label, Phase 3 study, compared TRC105 and pazopanib versus pazopanib alone in patients with advanced angiosarcoma, using an adaptive design with sample size re-estimation and population enrichment [52, 53]. The primary objective was to demonstrate superior progression-free survival (PFS) in the combination arm (TRC105 + pazopanib) compared to pazopanib alone. Due to early indications of higher tumour sensitivity to TRC105 in the cutaneous subgroup, patients were stratified by angiosarcoma subtype (cutaneous vs. non-cutaneous), with the option to restrict future enrolment to the cutaneous group.

5. Adaptations to patient allocation

There are two common types of adaptive randomization designs: covariate-adaptive randomization and response-adaptive randomization (RAR).

Covariate-adaptive randomization assigns patients to treatment groups based on baseline characteristics to ensure the balance between treatment groups and minimizing differences in prognostic covariates. Minimization is a well-known method for evenly distributing baseline characteristics [54]. The PATRICIA trial (PApilloma TRIal against Cancer In young Adults) used minimization to balance age ranges and study sites between treatment groups [55].

Response-adaptive randomization (RAR), on the other hand, is quite different and begins with equal randomization and adjusts the probability of assigning new participants to treatment arms based on accumulating outcomes. This method dynamically favours more effective treatments as data accumulate, increasing participants’ chances of receiving promising interventions. Robertson et al. [56] review the fundamental concepts and practical considerations for implementing RAR designs, including examples in non-vaccine settings.

6. Seamless Phase 2/3 adaptive design

A seamless Phase 2/3 design integrates the exploratory (Phase 2) and confirmatory (Phase 3) phases into a single trial. This reduces resource usage and timelines by eliminating the gap between phases and allows Phase 2 data to be included in the final analysis.

There are two key types of seamless designs: First, an operationally seamless Phase 2/3 design combines a Phase 2 dose-selection study and a Phase 3 confirmatory study within a single protocol, removing the pause between phases. Second, an inferentially seamless Phase 2/3 design goes a step further by incorporating Phase 2 data into the pivotal hypothesis testing and estimation, thereby reducing the sample size required for the Phase 3 portion [57].

Seamless Phase 2/3 designs are more commonly used in oncology but are gaining traction in vaccine development. Chen et al. [58] used a seamless Phase 2/3 design to develop a nine-valent HPV (9vHPV) vaccine, saving time and resources [59]. Yang et al. [60] discussed the application of seamless Phase 2/3 designs to expedite multi-valent vaccine development. The safety and immunogenicity of ChAdOx1 nCoV-19 vaccine was assessed in a single-blind, randomized Phase 2/3 trial [61].

7. Master protocols

Master protocols can be used where multiple treatments, subgroups of patients, or disease variants are tested simultaneously. The FDA provides guidance on Master Protocols [62], defining them as “a protocol designed with multiple sub-studies, which may have different objectives and involve coordinated efforts to evaluate one or more medical products in one or more diseases or conditions within the overall study structure”. Each sub-study includes the information and design features related to evaluation of a single product in a single disease, condition or disease subtype in the master protocol. Under this broad definition, master protocols encompass three main types of trials: umbrella, basket, and platform trials, each defined as follows by FDA Guidance [62]:

“*Umbrella trial*: This type of trial is designed to evaluate multiple medical products concurrently for a single disease or condition.

*Platform trial*: An ongoing trial structure used to evaluate multiple medical products for a disease or condition, with products being added or removed from the platform over time.

*Basket trial*: A trial designed to evaluate a single medical product for multiple diseases, conditions, or disease subtypes.”

Woodcock et al. [63] provide more detailed information on these trial types.

Platform trials have recently gained popularity, especially during the COVID-19 pandemic. The simultaneous assessment of multiple interventions and typical re-use of a single control arm for comparisons along with ability to add in further interventions after the trial has initiated means platform trials can be more efficient than sequential two-armed comparisons and simpler adaptive trials. Examples include the REMAP-CAP and RECOVERY trials, which tested multiple treatments for COVID-19, adding in new interventions over time [64, 65]. One notable platform vaccine trial is the WHO Solidarity Trial Vaccines (STV) study [66], an international initiative to evaluate the efficacy and safety of promising new COVID-19 vaccines.

Master protocols offer significant advantages through evaluating multiple research questions under one overarching protocol, but they come with increased costs in terms of time, resources, and planning. They require extensive infrastructure, greater coordination, and involvement of multiple stakeholders to agree on design, operations, and governance, unlike stand-alone trials. Despite the added complexity, master protocols can improve data quality, enhance trial efficiency, and, when designed effectively, can remain in use for years, accelerating the translation of laboratory innovations into clinical evaluation [63].

8. Adapting multiple design features

It is possible to integrate two or more adaptive design features. For instance, a group-sequential design may include a sample size re-estimation and/or adaptive randomization. Platform trials inherently involve multiple adaptive design elements. In practice, statistical inference for such multifaceted adaptive designs is often more challenging. The SHINE trial, assessing intensive versus standard hyperglycaemia treatment in acute ischemic stroke, features a group-sequential design, sample size re-estimation, and response-adaptive randomization [67].

Resources, including software, to support the implementation of different types of adaptive designs are available from the PANDA (Practical Adaptive and Novel Designs and Analysis toolkit) website [68].

9. Potential challenges

While adaptive designs offer potential significant benefits, such as shortening trial durations or ethical advantages of providing more patients with the better performing treatment or enabling more robust conclusions, they are generally more complex to design and conduct than traditional designs. Major regulatory agencies in Europe and the US have recently issued detailed guidelines on adaptive designs [31, 69] and are generally supportive of their use, particularly when the design is well-justified and concerns about controlling Type I error rates and bias are addressed [70, 71]. However, key considerations in planning adaptive designs include effective communication with stakeholders, securing funding, interpreting and reporting results, and addressing complex statistical issues—these are thoroughly discussed by Pallmann et al. [32].

## Bayesian approach in designing and analysing clinical trials

Traditionally, trials are designed and analysed using frequentist statistical methods, which begin with a hypothesis that is tested against a null hypothesis, leading to a *P*-value. The *P*-value indicates the probability of obtaining data at least as extreme as what was observed, assuming the null hypothesis is true. Established practice dichotomizes the *P*-value, leading to a binary decision: to reject or not reject the null hypothesis, resulting in a conclusion of either statistically significant or not.

In contrast, Bayesian methods estimate the probability that a hypothesis is true given the observed data, which can be more intuitive. Bayesian methods combine the observed data with prior knowledge about intervention effects, facilitating increases in efficiency and sequential learning. The prior information represents the likelihood of a treatment outcome before data are gathered, while the posterior probability is the updated likelihood after also considering the data collected during the trial.

Using informative priors in Bayesian methods can result in more precise inferences or enable reduced sample sizes, which is particularly important for rare diseases or hard-to-study populations [72]. To create informative priors, information can be borrowed from external data sources or prior elicitation techniques can be used [73].

Applying Bayesian methods in trial analysis can also enhance result interpretation [74]. The recently introduced Acceptability Curve Estimation using the Probability Above Threshold (ACCEPT) method [75] aims to improve and harmonize trial reporting. ACCEPT plots the probability of the true difference between treatments exceeding various “acceptability thresholds“, facilitating comparisons between trials with different designs and providing a more nuanced data interpretation.

Bayesian methods can be used for standard RCTs and any of the aforementioned adaptive designs. According to FDA guidance [31], Bayesian adaptive designs follow the same principles as adaptive designs without Bayesian features.

Since 1994, the methodological and ethical benefits of Bayesian approaches have been considered for designing late-phase trials, though they have not been widely adopted in practice [76]. Nowadays, advances in computational software have made it easier to employ Bayesian methods for designing and analysing clinical trials. However, more extensive simulations are typically needed to assess the operating characteristics of Bayesian designs.

While there are still limited publications on using Bayesian design in vaccine development and regulatory settings, their conceptual flexibility and ability to incorporate historical data offer a valuable alternative for modern vaccine trials. The original BNT162b2 vaccine study used Bayesian analysis, and results for the primary efficacy endpoint for both the interim and final analyses were reported through Bayesian inference [77].

## Using estimands in trial design

Whether a Bayesian or Frequentist approach is taken for analysis, a key issue in clinical trials is the need to target clear and meaningful treatment effects. To address this, in November 2019, the International Council for Harmonisation of Technical Requirements for Pharmaceuticals for Human (ICH) published a new framework for incorporating estimands into trial design (ICHE9(R1)) [78]. An estimand is defined as a precise description of the treatment effect that a trial aims to find out, different to the statistical method (estimator) used to compute the trial result (the estimate). By being clear about the targeted treatment effects during trial planning by specifying estimands this can then ensure an appropriate trial design, conduct and analysis method are employed to address what is of interest. The estimand framework is now adopted by regulators worldwide, meaning trialists should be defining estimands and stating these in their trial protocol. The 2023 EMA guidelines on clinical evaluation of vaccines [4] highlight how the agreed primary target of estimation (estimand) should be specified for vaccine trials as determined by the trial’s objective.

Defining an estimand requires specifying five attributes: (i) the patient population of interest, (ii) the treatment conditions being compared, (iii) the outcome/endpoint measure, (iv) handling of intercurrent events, and (v) the statistical summary measure. Intercurrent events are post-baseline events affecting outcome interpretation or existence, hence it is important to clarify what effect is being targeted with respect to these. In vaccine trials, typical intercurrent events are *not receiving the initial vaccine dose* and *not receiving subsequent vaccine doses* [4]. To estimate the effect of a vaccine in routine use, the estimand population would likely include all the eligible trial population who received at least one dose, and not receiving subsequent vaccine doses would be handled with a treatment policy strategy. This strategy considers missed vaccine doses as irrelevant and addresses efficacy regardless of full adherence. Alternatively, for estimating vaccine efficacy under full adherence the estimand population would be the eligible trial population who receive the full vaccine schedule, corresponding to using a principal stratum strategy.

By defining the primary estimand, a trial can be designed, conducted, and analysed using methods that align with the primary research question. A treatment policy strategy would include all randomized individuals who received at least one dose of the assigned treatment in the analysis population. In contrast, a principal stratum strategy, assuming no systematic differences between individuals who complete all vaccine doses in each randomized group, would include only subjects who received all doses in the analysis population [79].

Estimands are now being used in pivotal vaccine trials, for example the Pfizer COVID-19 vaccine Phase 2/3 trial [80]. The estimand primarily assessed in this trial was, the effect of prophylactic BNT162b2 against placebo (treatment conditions) in participants without evidence of COVID-19 infection before vaccination (population) who comply with the key protocol criteria (evaluable participants) at least 7 days after receipt of the second dose of study intervention (handling of intercurrent events using a principal stratum strategy) against confirmed COVID-19 occurring from 7 days after the second dose (outcome/endpoint), as 100 × (1 − IRR) [ratio of active vaccine to placebo incidence] (statistical summary measure).

Without specifying estimands, it has been found that most often it is unclear precisely what is being estimated in trials, potentially leading to misinterpretation or the use of inappropriate trial methods [81]. For a detailed primer on estimands and further examples, see Kahan et al [82].

## Advances in statistical analysis methods

Historically, trials have been analysed using simple statistical tests and unadjusted approaches that estimate the crude average effect of a treatment on an outcome without considering other influencing factors. Commonly used statistical tests included the *t*-test and ANOVA for continuous outcomes, the chi-square test for binary outcomes, and the log-rank test for time-to-event or survival outcomes.

While unadjusted analyses in individually randomized trials are unbiased on average, there are several reasons why covariate-adjusted approaches are preferable [83, 84]. EMA guideline [85] provides recommendations on which covariates should be adjusted for. Firstly, if covariates are used in the randomization process, such as through stratified randomization, adjusting for these covariates in the analysis is necessary to avoid incorrect Type I error rates and reductions in power [85, 86]. Secondly, adjusting for covariates not used in randomization may significantly increase the statistical power when these covariates are highly prognostic. Adjusting for the baseline value of a continuous primary outcome measure and covariates that have a strong or moderate association with the primary outcome generally improves the efficiency of the analysis [85].

There are various statistical methods for adjusting covariates, such as regression models, standardization and inverse-probability-of-treatment weighting [87], and the choice of method will depend on the nature of the covariate, outcome variable, and trial context.

Incorporating adaptive and innovative trial designs requires more advanced statistical approaches for both design and analysis. Simulation studies are typically used to estimate the required sample size for adaptive designs and to assess the trial’s operating characteristics, which is particularly true for Bayesian approaches. Conventional methods for estimating treatment effects at the end of a trial often lead to bias in many adaptive designs because they do not account for potential trial adaptations [88]. Various methods have been proposed to address this issue [89–91].

## Machine learning methods for the analysis of clinical trials

Whilst standard statistical methods used in RCTs typically estimate the average treatment effect (ATE) [92], meaning the overall effect of a treatment across all participants. Treatment response can often vary between individuals; some may benefit more than others. Estimating variation in treatment effects may consequently be important. This may be particularly important in vaccine trials as they often include large and diverse populations. Understanding variation in treatment effects may assist with a better understanding of the effect of a vaccine on the population of interest [93] especially when considering the waning of vaccine efficacy or when a null average effect is observed.

Traditionally, such variation is examined by assessing whether treatment effects differ significantly across defined subgroups of the population. This is achieved in practice by performing subgroup analyses which test single treatment-covariate interaction models for each covariate. However, this is limiting, relying on pre-defined subgroups and without considering interactions between multiple covariates and the treatment, it may not adequately describe subgroup differences. These analyses are also typically underpowered, and when a large number of subgroups are assessed, can increase the risk of chance findings [94]. Advances in machine learning methodology allow barriers associated with traditional subgroup analysis to be overcome. While machine learning algorithms were developed to predict outcomes, modifications have been made to target the estimation of variation in treatment effects, often referred to as heterogeneous treatment effects (HTEs). There are many emerging valid machine learning methods that can be used to estimate HTEs, such as meta-learners [95], targeted maximum likelihood estimation (TMLE) [96], causal forests (CFs) [97], and Bayesian additive regression trees (BARTs) [98]. These methods target estimating the conditional average treatment effect (CATE), that is, the treatment effect dependent on a set of baseline patient covariates in a single analysis. These newer machine learning models enable the exploration of higher-order interactions with the treatment effect, potentially leading to the identification of more detailed subpopulations that benefit from a particular treatment which may not have been considered or pre-defined using more traditional subgroup analyses.

Machine learning methods such as CFs have been used in secondary analyses of trials to explore variation in treatment effects. A re-analysis of the 65 trial, which evaluated the effect of a permissive hypotension strategy versus usual care on 90-day mortality for critically ill patients using CFs identified 10 potential subgroups with differing treatment responses defined by various combinations of five covariates (chronic hypertension, sepsis, duration of vasopressor use, age, and SOFA score). After validation in external datasets, these previously unconsidered subgroups could be used to target future research [99].

Chernozhukov et al. [100] developed generic machine learning tools to make inferences on HTEs, demonstrating their application to childhood vaccination strategies in India. Although there are many possible strategies for increasing immunization, combining multiple incentives is needed for effectiveness. This combination of incentives (local ambassadors, SMS reminders, and financial incentives) is more expensive to implement. Therefore, policymakers are interested in finding the groups that would benefit most from the combination of strategies. Their analysis suggested that vaccination incentives most impacted villages with the least pretreatment immunization.

Machine learning methods can also be used for other trial purposes. For example, machine learning algorithms can identify poorly performing sites and optimize site visit schedules. Algorithms can also be developed to improve recruitment strategies or evaluate the quality and completeness of reporting, which would be of great importance in a decentralized trial [101, 102].

## Safety analysis

The evaluation of safety in vaccine trials follows the same principles as those for other medicinal products [4]. However, because vaccines typically target healthy individuals, safety considerations are given more prominent emphasis in the evidence base. Vaccine safety is evaluated through routine collection of reactogenicity data (also called solicited adverse events) and clinical adverse event data. Reactogenicity refers to the set of adverse events resulting from the physical inflammatory response to the vaccination and includes localized events such as injection-site pain and systemic events such as fever or headache. Reactogenicity data are systematically collected from all participants for ~5–7 days after vaccination [103]. Like trials of other medicinal products, vaccine trials also collect unsolicited, self-reported adverse events; how long these are collected post-vaccine will depend on the vaccine’s characteristics and any prior evidence [4].

In therapy trials, adverse event data analysis typically relies on simplistic approaches that do not fully use the prospective, high-quality data collected, and known best practices in the analysis are often overlooked [104]. While efforts are being made to improve these practices, they predominantly focus on the analysis of prespecified safety outcomes [105]. Similarly, in vaccine studies, we see novel efforts to present systematically collected reactogenicity data. Visualization methods such as the stacked bar chart and, in more complicated scenarios with multiple treatment arms, radial graphs have been used to illustrate local and systemic reactions over the immediate post-vaccination period in COVID-19 vaccine trials [106, 107].

Nevertheless, like therapy trials, improvements in analysis practices are still needed. Greater consideration should be given to unsolicited adverse events at the design stage, specifying clear analysis plans to obtain the estimates of interest [108]. Principled analysis approaches that are accepted good practice for efficacy outcomes should also be applied to unsolicited adverse events. For example, using information on recurrent events rather than just presenting as those who experienced “at least one event”, appropriately accounting for varying follow-up times, and reducing information loss by retaining continuous outcomes in their natural form instead of dichotomizing to analyse as binary outcomes [109, 110]. Underpowered hypothesis tests and presenting *P*-values and confidence intervals as proxies for null hypotheses testing should also be avoided. Instead, the research question when analysing safety data should be reframed to focus on detecting safety signals for further investigation [104].

## Concluding remarks

This overview has highlighted recent developments and novel methodological and statistical approaches for the design and analysis of Phase 3 vaccine trials. Reflecting advancements in other medical fields [44, 111], we anticipate an increase in the use of adaptive designs in the vaccine trials, which offer ethical advantages and enable early conclusions if indicated. However, the suitability of an adaptive design should be carefully evaluated for each specific trial context. In particular, while we believe vaccine trials can significantly benefit from group-sequential designs, multi-arm multi-stage trials, population enrichment strategies, and platform trials, their effectiveness may be limited when the endpoint of interest takes too long to record, preventing adaptive changes from being implemented before the trial concludes [32, 112]. In extreme cases, all patients could be recruited before enough information is available from the assessed patients to make informed decisions. The use of adaptive designs or Bayesian methods also needs acceptance and familiarity among researchers, funders and sponsors. Conventional randomized controlled trials (RCTs) will continue to be appropriate in some settings, and researchers should consider the most suitable methodological approach for their specific objectives. Employing the estimand framework aligns trial design, conduct, and analysis with the primary research question, enhancing clarity and ensuring that trials address the most important questions. We advocate that trialists adopt this framework to improve the relevance and transparency of their studies.

In summary, while novel clinical trial designs and analysis methods offer significant potential for Phase 3 vaccine trials, it is crucial to invest time in understanding these methods and to implement changes thoughtfully to fully realize their benefits.

## Data Availability

The manuscript does not contain any original data, as it is a review.

## References

[1] Parrino J, Graham BS. Smallpox vaccines: past, present, and future. J Allergy Clin Immunol 2006, 118, 1320–6. doi: https://doi.org/10.1016/j.jaci.2006.09.037 17157663 PMC9533821

[2] U. S. Centers for Disease Control and Prevention. Ten great public health achievements -- United States, 1900-1999. 1999. https://www.cdc.gov/mmwr/preview/mmwrhtml/00056796.htm 10227303

[3] Conti AA. Vaccination through time: from the first smallpox vaccine to current vaccination campaigns against the COVID-19 pandemic. Acta Biomed 2021, 92, e2021453. doi: https://doi.org/10.23750/abm.v92iS6.12211 34739474 PMC8851018

[4] Committee for Medicinal Products for Human Use [CHMP]. Guideline on clinical evaluation of vaccines. 2023. https://www.ema.europa.eu/en/documents/scientific-guideline/guideline-clinical-evaluation-vaccines-revision-1_en.pdf

[5] Verdecia M, Kokai-Kun JF, Kibbey M, Acharya S, Venema J, Atouf F. COVID-19 vaccine platforms: delivering on a promise? Hum Vaccin Immunother 2021, 17, 2873–93. doi: https://doi.org/10.1080/21645515.2021.1911204 34033528 PMC8381795

[6] Heaton PM. Challenges of developing novel vaccines with particular global health importance. *Front Immunol* 2020, 11, 517290. doi: https://doi.org/10.3389/fimmu.2020.517290 33162972 PMC7591467

[7] Madewell ZJ, Dean NE, Berlin JA, Coplan PM, Davis KJ, Struchiner CJ, et al Challenges of evaluating and modelling vaccination in emerging infectious diseases. Epidemics 2021, 37, 100506. doi: https://doi.org/10.1016/j.epidem.2021.100506 34628108 PMC8491997

[8] Senn S. The design and analysis of vaccine trials for COVID-19 for the purpose of estimating efficacy. Pharm Stat 2022, 21, 790–807. doi: https://doi.org/10.1002/pst.2226 35819115 PMC9350415

[9] Day S. Dictionary for clinical trials. 2nd ed. Chichester: John Wiley & Sons, 2007.

[10] Agresti A, Franklin C, Klingenberg B. Statistics: the art and science of learning from data. Global edition, 4th ed. Harlow: Pearson Education, 2018.

[11] Saleh A, Qamar S, Tekin A, Singh R, Kashyap R. Vaccine development throughout history. Cureus 2021, 13, p. e16635. doi: https://doi.org/10.7759/cureus.16635 34462676 PMC8386248

[12] Bhatt A. Evolution of clinical research: a history before and beyond James Lind. Perspect Clin Res 2010, 1, 6–10. 21829774 PMC3149409

[13] Meldrum M. “A calculated risk”: the Salk polio vaccine field trials of 1954. BMJ 1998, 317, 1233–6. doi: https://doi.org/10.1136/bmj.317.7167.1233 9794869 PMC1114166

[14] Ebola ça Suffit Ring Vaccination Trial Consortium. The ring vaccination trial: a novel cluster randomised controlled trial design to evaluate vaccine efficacy and effectiveness during outbreaks, with special reference to Ebola. BMJ 2015, 351, h3740. doi: https://doi.org/10.1136/bmj.h3740 26215666 PMC4516343

[15] Henao-Restrepo AM, Camacho A, Longini IM, Watson CH, Edmunds WJ, Egger M, et al Efficacy and effectiveness of an rVSV-vectored vaccine in preventing Ebola virus disease: final results from the Guinea ring vaccination, open-label, cluster-randomised trial (Ebola Ça Suffit!). Lancet 2017, 389, 505–18. doi: https://doi.org/10.1016/S0140-6736(16)32621-6 28017403 PMC5364328

[16] Dean NE, Longini IM. The ring vaccination trial design for the estimation of vaccine efficacy and effectiveness during infectious disease outbreaks. Clin Trials 2022, 19, 402–6. doi: https://doi.org/10.1177/17407745211073594 35057647 PMC9300768

[17] Samai M, Seward JF, Goldstein ST, Mahon BE, Lisk DR, Widdowson M-A, et al; STRIVE Study Team. The Sierra Leone trial to introduce a vaccine against Ebola: an evaluation of rVSV∆ G-ZEBOV-GP vaccine tolerability and safety during the West Africa Ebola outbreak. J Infect Dis 2018, 217, S6–S15. doi: https://doi.org/10.1093/infdis/jiy020 29788345 PMC5961340

[18] Piszczek J, Partlow E. Stepped-wedge trial design to evaluate Ebola treatments. Lancet Infect Dis 2015, 15, 762–3. doi: https://doi.org/10.1016/S1473-3099(15)00078-X 26122441

[19] Hemming K, Haines TP, Chilton PJ, Girling AJ, Lilford RJ. The stepped wedge cluster randomised trial: rationale, design, analysis, and reporting. BMJ 2015, 350, h391. doi: https://doi.org/10.1136/bmj.h391 25662947

[20] U. S. Centers for Disease Control and Prevention. How vaccines are developed and approved for use. https://www.cdc.gov/vaccines/basics/how-developed-approved.html#:~:text=The%20Advisory%20Committee%20on%20Immunization,that%20are%20approved%20by%20FDA

[21] Han S. Clinical vaccine development. Clin Exp Vaccine Res 2015, 4, 46–53. doi: https://doi.org/10.7774/cevr.2015.4.1.46 25648742 PMC4313108

[22] Singh K, Mehta S. The clinical development process for a novel preventive vaccine: an overview. J Postgrad Med 2016, 62, 4–11. doi: https://doi.org/10.4103/0022-3859.173187 26732191 PMC4944327

[23] Tentori K, Passerini A, Timberlake B, Pighin S. The misunderstanding of vaccine efficacy. Soc Sci Med 2021, 289, 114273. doi: https://doi.org/10.1016/j.socscimed.2021.114273 34619632 PMC8314794

[24] Stern AM, Markel H. The history of vaccines and immunization: familiar patterns, new challenges. Health Aff (Millwood) 2005, 24, 611–21. doi: https://doi.org/10.1377/hlthaff.24.3.611 15886151

[25] Farrington CP, Miller E. Vaccine trials. Mol Biotechnol 2001, 17, 43–58. doi: https://doi.org/10.1385/MB:17:1:43 11280930

[26] Charles P, Giraudeau B, Dechartres A, Baron G, Ravaud P. Reporting of sample size calculation in randomised controlled trials: review. BMJ 2009, 338, b1732. doi: https://doi.org/10.1136/bmj.b1732 19435763 PMC2680945

[27] Stallard N, Hampson L, Benda N, Brannath W, Burnett T, Friede T, et al Efficient adaptive designs for clinical trials of interventions for COVID-19. Stat Biopharm Res 2020, 12, 483–97. doi: https://doi.org/10.1080/19466315.2020.1790415 34191981 PMC8011600

[28] Brueckner M, Titman A, Jaki T, Rojek A, Horby P. Performance of different clinical trial designs to evaluate treatments during an epidemic. PLoS One 2018, 13, e0203387. doi: https://doi.org/10.1371/journal.pone.0203387 30204799 PMC6133355

[29] Singh JA, Kochhar S, Wolff J, Atuire C, Bhan A, Emanuel E, et al WHO guidance on COVID-19 vaccine trial designs in the context of authorized COVID-19 vaccines and expanding global access: ethical considerations. Vaccine 2022, 40, 2140–9. doi: https://doi.org/10.1016/j.vaccine.2022.02.038 35248422 PMC8882397

[30] Liu M, Li Q, Lin J, Lin Y, Hoffman E. Innovative trial designs and analyses for vaccine clinical development. Contemp Clin Trials 2021, 100, 106225. doi: https://doi.org/10.1016/j.cct.2020.106225 33227451 PMC7834363

[31] U. S. Food and Drug Administration [FDA]. Adaptive designs for clinical trials of drugs and biologics: guidance for industry. 2019. https://www.fda.gov/regulatory-information/search-fda-guidance-documents/adaptive-design-clinical-trials-drugs-and-biologics-guidance-industry

[32] Pallmann P, Bedding AW, Choodari-Oskooei B, Dimairo M, Flight L, Hampson LV, et al Adaptive designs in clinical trials: why use them, and how to run and report them. BMC Med 2018, 16, 29. doi: https://doi.org/10.1186/s12916-018-1017-7 29490655 PMC5830330

[33] Bhatt DL, Mehta C. Adaptive designs for clinical trials. N Engl J Med 2016, 375, 65–74. doi: https://doi.org/10.1056/NEJMra1510061 27406349

[34] Le Tourneau C, Lee JJ, Siu LL. Dose escalation methods in phase I cancer clinical trials. J Natl Cancer Inst 2009, 101, 708–20. doi: https://doi.org/10.1093/jnci/djp079 19436029 PMC2684552

[35] Hoering A, LeBlanc M, Crowley J. Seamless phase I-II trial design for assessing toxicity and efficacy for targeted agents. Clin Cancer Res 2011, 17, 640–6. doi: https://doi.org/10.1158/1078-0432.CCR-10-1262 21135145 PMC4391513

[36] O’Brien PC, Fleming TR. A multiple testing procedure for clinical trials. Biometrics 1979, 35, 549–56. doi: https://doi.org/10.2307/2530245 497341

[37] Pocock SJ. Group sequential methods in the design and analysis of clinical trials. Biometrika 1977, 64, 191. doi: https://doi.org/10.2307/2335684

[38] Flynn NM, Forthal DN, Harro CD, Judson FN, Mayer KH, Para MF; rgp120 HIV Vaccine Study Group. Placebo-controlled phase 3 trial of a recombinant glycoprotein 120 vaccine to prevent HIV-1 infection. J Infect Dis 2005, 191, 654–65. doi: https://doi.org/10.1086/428404 15688278

[39] Magirr D, Jaki T, Whitehead J. A generalized Dunnett test for multi-arm multi-stage clinical studies with treatment selection. Biometrika 2012, 99, 494–501. doi: https://doi.org/10.1093/biomet/ass002

[40] Wason J, Magirr D, Law M, Jaki T. Some recommendations for multi-arm multi-stage trials. Stat Methods Med Res 2016, 25, 716–27. doi: https://doi.org/10.1177/0962280212465498 23242385 PMC4843088

[41] Millen GC, Yap C. Adaptive trial designs: what are multiarm, multistage trials? Arch Dis Child Educ Pract Ed 2020, 105, 376–8. doi: https://doi.org/10.1136/archdischild-2019-317826 31662314

[42] Wu J, Li Y, Zhu L. Group sequential multi-arm multi-stage trial design with treatment selection. Stat Med 2023, 42, 1480–91. doi: https://doi.org/10.1002/sim.9682 36808736

[43] Moore CL, Stöhr W, Crook AM, Richert L, Leliévre J-D, Pantaleo G, et al Multi-arm, multi-stage randomised controlled trials for evaluating therapeutic HIV cure interventions. The Lancet HIV 2019, 6, e334–40. doi: https://doi.org/10.1016/S2352-3018(19)30082-7 31047670

[44] Noor NM, Love SB, Isaacs T, Kaplan R, Parmar MKB, Sydes MR. Uptake of the multi-arm multi-stage (MAMS) adaptive platform approach: a trial-registry review of late-phase randomised clinical trials. BMJ Open 2022, 12, e055615. doi: https://doi.org/10.1136/bmjopen-2021-055615 PMC8915371 35273052

[45] Mehta C, Bhingare A, Liu L, Senchaudhuri P. Optimal adaptive promising zone designs. Stat Med 2022, 41, 1950–70. doi: https://doi.org/10.1002/sim.9339 35165917

[46] Knobel D, Odita CI, Conan A, Barry D, Smith-Anthony M, Battice J, et al Non-specific effects of rabies vaccine on the incidence of common infectious disease episodes: study protocol for a randomized controlled trial. Trials 2020, 21, 534. doi: https://doi.org/10.1186/s13063-020-04467-z 32546199 PMC7296525

[47] Cui L, Hung HMJ, Wang SJ. Modification of sample size in group sequential clinical trials. Biometrics 1999, 55, 853–7. doi: https://doi.org/10.1111/j.0006-341x.1999.00853.x 11315017

[48] Denne JS. Sample size recalculation using conditional power. Stat Med 2001, 20, 2645–60. doi: https://doi.org/10.1002/sim.734 11523074

[49] Gao P, Ware JH, Mehta C. Sample size re-estimation for adaptive sequential design in clinical trials. J Biopharm Stat 2008, 18, 1184–96. doi: https://doi.org/10.1080/10543400802369053 18991116

[50] Mehta CR, Pocock SJ. Adaptive increase in sample size when interim results are promising: a practical guide with examples. Stat Med 2011, 30, 3267–84. doi: https://doi.org/10.1002/sim.4102 22105690

[51] Su S-C, Li X, Zhao Y, Chan ISF. Population-enrichment adaptive design strategy for an event-driven vaccine efficacy trial. Stat Biosci 2018, 10, 357–70. doi: https://doi.org/10.1007/s12561-017-9202-3

[52] Jones RL, Ravi V, Brohl AS, Chawla S, Ganjoo KN, Italiano A, et al Efficacy and safety of TRC105 plus pazopanib vs pazopanib alone for treatment of patients with advanced angiosarcoma: a randomized clinical trial. JAMA Oncol. 2022, 8, 740–7. doi: https://doi.org/10.1001/jamaoncol.2021.3547 35357396 PMC8972152

[53] Mehta CR, Liu L, Theuer C. An adaptive population enrichment phase III trial of TRC105 and pazopanib versus pazopanib alone in patients with advanced angiosarcoma (TAPPAS trial). Ann. Oncol. 2019, 30, 103–8. doi: https://doi.org/10.1093/annonc/mdy464 30357394 PMC6336002

[54] Pocock SJ, Simon R. Sequential treatment assignment with balancing for prognostic factors in the controlled clinical trial. Biometrics 1975, 31, 103–15. doi: https://doi.org/10.2307/2529712 1100130

[55] Lehtinen M, Paavonen J, Wheeler CM, Jaisamrarn U, Garland SM, Castellsagué X, et al; HPV PATRICIA Study Group. Overall efficacy of HPV-16/18 AS04-adjuvanted vaccine against grade 3 or greater cervical intraepithelial neoplasia: 4-year end-of-study analysis of the randomised, double-blind PATRICIA trial. Lancet Oncol 2012, 13, 89–99. doi: https://doi.org/10.1016/S1470-2045(11)70286-8 22075171

[56] Robertson DS, Lee KM, López-Kolkovska BC, Villar SS. Response-adaptive randomization in clinical trials: from myths to practical considerations. Stat Sci 2023, 38, 185–208. doi: https://doi.org/10.1214/22-STS865 37324576 PMC7614644

[57] Cuffe RL, Lawrence D, Stone A, Vandemeulebroecke M. When is a seamless study desirable? Case studies from different pharmaceutical sponsors. Pharm Stat 2014, 13, 229–37. doi: https://doi.org/10.1002/pst.1622 24891148

[58] Chen YH, Gesser R, Luxembourg A. A seamless phase IIB/III adaptive outcome trial: design rationale and implementation challenges. Clin Trials 2015, 12, 84–90. doi: https://doi.org/10.1177/1740774514552110 25278227

[59] Miller E, Gallo P, He W, Kammerman LA, Koury K, Maca J, et al DIA’s Adaptive Design Scientific Working Group (ADSWG): best practices case studies for “Less Well-understood” adaptive designs. Ther. Innov. Regul. Sci. 2017, 51, 77–88. doi: https://doi.org/10.1177/2168479016665434 30235997

[60] Yang JY, Li GC, Yuan Y. Accelerate vaccine development using seamless phase 2/3 trial designs. Expert Rev Vaccines 2024, 23, 523–34. doi: https://doi.org/10.1080/14760584.2024.2348612 38682812

[61] Ramasamy MN, Minassian AM, Ewer KJ, Flaxman AL, Folegatti PM, Owens DR, et al; Oxford COVID Vaccine Trial Group. Safety and immunogenicity of ChAdOx1 nCoV-19 vaccine administered in a prime-boost regimen in young and old adults (COV002): a single-blind, randomised, controlled, phase 2/3 trial. The Lancet 2020, 396, 1979–93. doi: https://doi.org/10.1016/S0140-6736(20)32466-1 PMC7674972 33220855

[62] U. S. Food and Drug Administration [FDA]. Master Protocols for Drug and Biological Product Development: Guidance for Industry. 2023. https://www.fda.gov/regulatory-information/search-fda-guidance-documents/master-protocols-drug-and-biological-product-development

[63] Woodcock J, LaVange LM. Master protocols to study multiple therapies, multiple diseases, or both. N Engl J Med 2017, 377, 62–70. doi: https://doi.org/10.1056/NEJMra1510062 28679092

[64] REMAP-CAP Investigators. Randomized, embedded, multifactorial adaptive platform trial for community- acquired pneumonia (REMAP-CAP). 2016. https://www.remapcap.org/10.1513/AnnalsATS.202003-192SD PMC7328186 32267771

[65] RECOVERY Collaborative Group. Randomised evaluation of COVID-19 therapy (RECOVERY). 2020. https://www.recoverytrial.net/#:~:text=RECOVERY%20is%20an%20international%20clinical,hospital%20with%20COVID%2D19%20pneumonia

[66] World Health Organization [WHO]. Solidarity trial vaccines. 2021. https://www.who.int/emergencies/diseases/novel-coronavirus-2019/global-research-on-novel-coronavirus-2019-ncov/solidarity-trial-of-covid-19-vaccines

[67] Johnston KC, Bruno A, Pauls Q, Hall CE, Barrett KM, Barsan W, et al; Neurological Emergencies Treatment Trials Network and the SHINE Trial Investigators. Intensive vs standard treatment of hyperglycemia and functional outcome in patients with acute ischemic stroke: the SHINE randomized clinical trial. JAMA 2019, 322, 326–35. doi: https://doi.org/10.1001/jama.2019.9346 31334795 PMC6652154

[68] PANDA Toolkit Team. A practical adaptive & novel designs and analysis toolkit. PANDA. https://panda.shef.ac.uk/

[69] Committee for Medicinal Products for Human Use [CHMP]. Reflection paper on methodological issues in confirmatory clinical trials planned with an adaptive design. European Medicines Agency, 2007. https://www.ema.europa.eu/en/documents/scientific-guideline/reflection-paper-methodological-issues-confirmatory-clinical-trials-planned-adaptive-design_en.pdf

[70] Gaydos B, Koch A, Miller F, Posch M, Vandemeulebroecke M, Wang S-J. Perspective on adaptive designs: 4 years European Medicines Agency reflection paper, 1 year draft US FDA guidance–where are we now? Clin Investig. 2012, 2, 235–40. doi: https://doi.org/10.4155/cli.12.5

[71] Elsäßer A, Regnstrom J, Vetter T, Koenig F, Hemmings RJ, Greco M, et al Adaptive clinical trial designs for European marketing authorization: a survey of scientific advice letters from the European Medicines Agency. Trials 2014, 15, 1–10. doi: https://doi.org/10.1186/1745-6215-15-383 25278265 PMC4196072

[72] Siddique J, Aghabazaz Z. Prior ground: selection of prior distributions when analyzing clinical trial data using Bayesian methods. NEJM Evidence 2023, 2, EVIDe2300250. doi: https://doi.org/10.1056/EVIDe2300250 38320533 PMC11197078

[73] Dallow N, Best N, Montague TH. Better decision making in drug development through adoption of formal prior elicitation. Pharm Stat 2018, 17, 301–16. doi: https://doi.org/10.1002/pst.1854 29603614

[74] Muehlemann N, Zhou T, Mukherjee R, Hossain MI, Roychoudhury S, Russek-Cohen E. A *tutorial on modern Bayesian methods in clinical trials*. Ther Innov Regul Sci 2023, 57, 402–16. doi: https://doi.org/10.1007/s43441-023-00515-3 37081374 PMC10117244

[75] Clements MN, White IR, Copas AJ, Cornelius V, Cro S, Dunn DT, et al Improving clinical trial interpretation with ACCEPT analyses. NEJM Evidence 2022, 1, evidctw2200018. doi: https://doi.org/10.1056/EVIDctw2200018 35965674 PMC7613267

[76] Spiegelhalter DJ, Freedman LS, Parmar MKB. Bayesian approaches to randomized trials. J R Statist Soc A 1994, 157, 357. doi: https://doi.org/10.2307/2983527

[77] Polack FP, Thomas SJ, Kitchin N, Absalon J, Gurtman A, Lockhart S, et al; C4591001 Clinical Trial Group. Safety and efficacy of the BNT162b2 mRNA Covid-19 vaccine. N Engl J Med 2020, 383, 2603–15. doi: https://doi.org/10.1056/NEJMoa2034577 33301246 PMC7745181

[78] International Council for Harmonisation of Technical Requirements for Pharmaceuticals for Human Use [ICH]. Addendum on estimands and sensitivity analysis in clinical trials to the guideline on statistical principles for clinical trials E9(R1). European medicines Agency, 2019. https://www.ema.europa.eu/en/documents/scientific-guideline/ich-e9-r1-addendum-estimands-and-sensitivity-analysis-clinical-trials-guideline-statistical-principles-clinical-trials-step-5_en.pdf

[79] Kahan BC, White IR, Edwards M, Harhay MO. Using modified intention-to-treat as a principal stratum estimator for failure to initiate treatment. Clin Trials 2023, 20, 269–75. doi: https://doi.org/10.1177/17407745231160074 36916466 PMC10262320

[80] Pfizer B. A Phase 1/2/3, placebo-controlled, randomized, observer-blind, dose-finding study to evaluate the safety, tolerability, immunogenicity, and efficacy of SARS-CoV-2 RNA vaccine candidates against COVID-19 in healthy individuals. 2020. https://data.parliament.uk/DepositedPapers/Files/DEP2023-0138/Clinical_Study_Report_Part_1.pdf

[81] Cro S, Kahan BC, Rehal S, Chis Ster A, Carpenter JR, White IR, et al Evaluating how clear the questions being investigated in randomised trials are: systematic review of estimands. BMJ 2022, 378, e070146. doi: https://doi.org/10.1136/bmj-2022-070146 35998928 PMC9396446

[82] Kahan BC, Hindley J, Edwards M, Cro S, Morris TP. The estimands framework: a primer on the ICH E9(R1) addendum. BMJ 2024, 384, e076316. doi: https://doi.org/10.1136/bmj-2023-076316 38262663 PMC10802140

[83] Kahan BC, Rushton H, Morris TP, Daniel RM. A comparison of methods to adjust for continuous covariates in the analysis of randomised trials. BMC Med Res Methodol 2016, 16, 42. doi: https://doi.org/10.1186/s12874-016-0141-3 27068456 PMC4827223

[84] Tackney MS, Morris T, White I, Leyrat C, Diaz-Ordaz K, Williamson E. A comparison of covariate adjustment approaches under model misspecification in individually randomized trials. Trials 2023, 24, 14. doi: https://doi.org/10.1186/s13063-022-06967-6 36609282 PMC9817411

[85] Committee for Medicinal Products for Human Use [CHMP]. Guideline on Adjustment for Baseline Covariates in Clinical Trials. European Medicine Agency, 2015. https://www.ema.europa.eu/en/documents/scientific-guideline/guideline-adjustment-baseline-covariates-clinical-trials_en.pdf

[86] Kahan BC, Morris TP. Improper analysis of trials randomised using stratified blocks or minimisation. Stat Med 2012, 31, 328–40. doi: https://doi.org/10.1002/sim.4431 22139891

[87] Morris TP, Walker AS, Williamson EJ, White IR. Planning a method for covariate adjustment in individually randomised trials: a practical guide. Trials 2022, 23, 328. doi: https://doi.org/10.1186/s13063-022-06097-z 35436970 PMC9014627

[88] Robertson DS, Choodari-Oskooei B, Dimairo M, Flight L, Pallmann P, Jaki T. Point estimation for adaptive trial designs I: a methodological review. Stat Med 2023, 42, 122–45. doi: https://doi.org/10.1002/sim.9605 36451173 PMC7613995

[89] Robertson DS, Prevost AT, Bowden J. Unbiased estimation in seamless phase II/III trials with unequal treatment effect variances and hypothesis-driven selection rules. Stat Med 2016, 35, 3907–22. doi: https://doi.org/10.1002/sim.6974 27103068 PMC5026174

[90] Whitehead J, Desai Y, Jaki T. Estimation of treatment effects following a sequential trial of multiple treatments. Stat Med 2020, 39, 1593–609. doi: https://doi.org/10.1002/sim.8497 32207166 PMC7217198

[91] Choodari-Oskooei B, Parmar MKB, Royston P, Bowden J. Impact of lack-of-benefit stopping rules on treatment effect estimates of two-arm multi-stage (TAMS) trials with time to event outcome. Trials 2013, 14, 23. doi: https://doi.org/10.1186/1745-6215-14-23 23343147 PMC3599134

[92] Lipkovich I, Svensson D, Ratitch B, Dmitrienko A. Overview of modern approaches for identifying and evaluating heterogeneous treatment effects from clinical data. Clin Trials 2023, 20, 380–93. doi: https://doi.org/10.1177/17407745231174544 37203150

[93] Nikas A, Ahmed H, Zarnitsyna VI. Competing heterogeneities in vaccine effectiveness estimation. Vaccines 2023, 11, 1312. doi: https://doi.org/10.3390/vaccines11081312 37631880 PMC10458793

[94] Fingerhut A, Uranues S, Dziri C, Ma J, Vernerey D, Kurihara H, et al Interaction analysis of subgroup effects in randomized trials: the essential methodological points. Sci Rep 2024, 14, 12619. doi: https://doi.org/10.1038/s41598-024-62896-1 38824173 PMC11144206

[95] Künzel SR, Sekhon JS, Bickel PJ, Yu B. Metalearners for estimating heterogeneous treatment effects using machine learning. Proc Natl Acad Sci USA 2019, 116, 4156–65. doi: https://doi.org/10.1073/pnas.1804597116 30770453 PMC6410831

[96] Schuler MS, Rose S. Targeted maximum likelihood estimation for causal inference in observational studies. Am J Epidemiol 2017, 185, 65–73. doi: https://doi.org/10.1093/aje/kww165 27941068

[97] Athey S, Wager S. Estimating treatment effects with causal forests: an application. Obs. Stud 2019, 5, 37–51. doi: https://doi.org/10.1353/obs.2019.0001

[98] Hill J, Linero A, Murray J. Bayesian additive regression trees: a review and look forward. Annu Rev Stat Appl 2020, 7, 251–78. doi: https://doi.org/10.1146/annurev-statistics-031219-041110

[99] Sadique Z, Grieve R, Diaz-Ordaz K, Mouncey P, Lamontagne F, O'Neill S. A machine-learning approach for estimating subgroup- and individual-level treatment effects: an illustration using the 65 trial. Med Decis Making 2022, 42, 923–36. doi: https://doi.org/10.1177/0272989X221100717 35607982 PMC9459357

[100] Chernozhukov V, Demirer M, Duflo E, Fernández-Val I. Generic machine learning inference on heterogeneous treatment effects in randomized experiments, with an application to immunization in India. NBER Working Paper 2018, No. 24678, 1–69. doi: https://doi.org/10.3386/W24678

[101] Chopra H, Annu, Shin DK, Munjal K, Priyanka, Dhama K, et al Revolutionizing clinical trials: the role of AI in accelerating medical breakthroughs. Int J Surg 2023, 109, 4211–20. doi:10.1097/js9.0000000000000705 38259001 PMC10720846

[102] Feng J, Phillips RV, Malenica I, Bishara A, Hubbard AE, Celi LA, et al Clinical artificial intelligence quality improvement: towards continual monitoring and updating of AI algorithms in healthcare. NPJ Digital Med 2022, 5, 66. doi: https://doi.org/10.1038/s41746-022-00611-y PMC9156743 35641814

[103] Hervé C, Laupèze B, Del Giudice G, Didierlaurent AM, Tavares Da Silva F. The how’s and what’s of vaccine reactogenicity. NPJ Vaccines 2019, 4, 39. doi: https://doi.org/10.1038/s41541-019-0132-6 31583123 PMC6760227

[104] Cornelius VR, Phillips R. Improving the analysis of adverse event data in randomized controlled trials. J Clin Epidemiol 2022, 144, 185–92. doi: https://doi.org/10.1016/j.jclinepi.2021.12.023 34954021

[105] Stegherr R, Beyersmann J, Jehl V, Rufibach K, Leverkus F, Schmoor C, et al Survival analysis for AdVerse events with VarYing follow-up times (SAVVY): Rationale and statistical concept of a meta-analytic study. Biomed J 2021, 63, 650–70. doi: https://doi.org/10.1002/bimj.201900347 33145854

[106] Munro APS, Janani L, Cornelius V, Aley PK, Babbage G, Baxter D, et al; COV-BOOST study group. Safety and immunogenicity of seven COVID-19 vaccines as a third dose (booster) following two doses of ChAdOx1 nCov-19 or BNT162b2 in the UK (COV-BOOST): a blinded, multicentre, randomised, controlled, phase 2 trial. Lancet 2021, 398, 2258–76. doi: https://doi.org/10.1016/S0140-6736(21)02717-3 34863358 PMC8639161

[107] Oda Y, Kumagai Y, Kanai M, Iwama Y, Okura I, Minamida T, et al Immunogenicity and safety of a booster dose of a self-amplifying RNA COVID-19 vaccine (ARCT-154) versus BNT162b2 mRNA COVID-19 vaccine: a double-blind, multicentre, randomised, controlled, phase 3, non-inferiority trial. Lancet Infect Dis 2024, 24, 351–60. doi: https://doi.org/10.1016/S1473-3099(23)00650-3 38141632

[108] Xia HA, Jiang Q. Statistical evaluation of drug safety data. Ther Innov Regul Sci 2014, 48, 109–20. doi: https://doi.org/10.1177/2168479013510917 30231415

[109] Phillips R, Cornelius V. Future directions of research into harms in randomised controlled trials. BMJ 2023, 381, 926. doi: https://doi.org/10.1136/bmj.p926 37094837

[110] Rufibach K, Beyersmann J, Friede T, Schmoor C, Stegherr R. Survival analysis for AdVerse events with VarYing follow-up times (SAVVY): summary of findings and assessment of existing guidelines. Trials 2024, 25, 353. doi: https://doi.org/10.1186/s13063-024-08186-7 38822392 PMC11143657

[111] PRACTICAL, PANTHER, TRAITS, INCEPT, and REMAP-CAP Investigators. The rise of adaptive platform trials in critical care. Am J Respir Crit Care Med 2024, 209, 491–6. doi: https://doi.org/10.1164/rccm.202401-0101CP 38271622 PMC10919116

[112] Jaki T, Wason JM. Multi-arm multi-stage trials can improve the efficiency of finding effective treatments for stroke: a case study. BMC Cardiovasc Disord 2018, 18, 1–8. doi: https://doi.org/10.1186/s12872-018-0956-4 30482176 PMC6260683

